# Imaging PPG for In Vivo Human Tissue Perfusion Assessment during Surgery

**DOI:** 10.3390/jimaging8040094

**Published:** 2022-03-31

**Authors:** Marco Lai, Stefan D. van der Stel, Harald C. Groen, Mark van Gastel, Koert F. D. Kuhlmann, Theo J. M. Ruers, Benno H. W. Hendriks

**Affiliations:** 1 IGT & US Devices & Systems, Philips Research, High Tech, Campus 34, 5656 AE Eindhoven, The Netherlands; marco.lai@philips.com (M.L.); benno.hendriks@philips.com (B.H.W.H.); 2 Department of Electrical Engineering, Eindhoven University of Technology, 5600 MB Eindhoven, The Netherlands; mark.van.gastel@philips.com; 3 Faculty TNW, Group Nanobiophysics, Twente University, Drienorlaan 5, 7522 NB Enschede, The Netherlands; t.ruers@nki.nl; 4 Department of Surgery, The Netherlands Cancer Institute—Antoni van Leeuwenhoek, Plesmanlaan 121, Postbus 90203, 1066 CX Amsterdam, The Netherlands; h.groen@nki.nl (H.C.G.); k.kuhlmann@nki.nl (K.F.D.K.); 5 Patient Care & Monitoring, Philips Research, High Tech Campus 34, 5656 AE Eindhoven, The Netherlands; 6 Biomedical Engineering, Delft University of Technology, Mekelweg 5, 2628 CD Delft, The Netherlands

**Keywords:** imaging photoplethysmography, iPPG, intraoperative perfusion assessment, intestinal surgery, optical technology

## Abstract

Surgical excision is the golden standard for treatment of intestinal tumors. In this surgical procedure, inadequate perfusion of the anastomosis can lead to postoperative complications, such as anastomotic leakages. Imaging photoplethysmography (iPPG) can potentially provide objective and real-time feedback of the perfusion status of tissues. This feasibility study aims to evaluate an iPPG acquisition system during intestinal surgeries to detect the perfusion levels of the microvasculature tissue bed in different perfusion conditions. This feasibility study assesses three patients that underwent resection of a portion of the small intestine. Data was acquired from fully perfused, non-perfused and anastomosis parts of the intestine during different phases of the surgical procedure. Strategies for limiting motion and noise during acquisition were implemented. iPPG perfusion maps were successfully extracted from the intestine microvasculature, demonstrating that iPPG can be successfully used for detecting perturbations and perfusion changes in intestinal tissues during surgery. This study provides proof of concept for iPPG to detect changes in organ perfusion levels.

## 1. Introduction

Impaired perfusion of tissue in the anastomotic region in gastrointestinal surgery is a major contributor to postoperative complications such as anastomotic leakage or tissue necrosis [1]. These complications can result in the necessity of surgical revision, followed by increased morbidity and mortality, extended hospital stay, and increased health care costs [2,3]. Thus, adequate tissue perfusion is essential for a successful surgical outcome and postoperative recovery. For intestinal cancers, the part of the intestine where the tumor resides is excised, after which the proximal and distal ends of the intestine are anastomosed to restore the continuity of the intestine [4]. Presently, surgeons perform several clinical checks after re-establishing blood flow. These checks consist of examination of the color of the tissue and identification of palpable pulsations. However, these checks are mainly based on the surgeons’ clinical experience, and thus may lead to misinterpretations [5]. For this reason, an objective and real-time feedback of perfusion during surgery is desirable to minimize complications caused by inadequate perfusion and adequately predict postoperative wound healing and organ function. 

Several techniques are being researched to objectively assess tissue perfusion intra-operatively, such as fluorescence imaging, laser Doppler and hyperspectral imaging (HSI) [6,7,8]. The main limitations of those techniques are the dependence of indocyanine green (ICG) injections (fluorescence imaging), the limited field of view or disruption of the analysis due to intrinsic or extrinsic motions (laser Doppler). Thus, the challenge for a non-invasive technique that provides real-time feedback about the perfusion status of tissue during surgery still remains.

Photoplethysmography (PPG) is a simple and low-cost optical technique used non-invasively to measure blood-volume changes that occur in the microvascular tissue bed at the skin surface [9,10,11,12,13]. The basic configuration of this contact PPG technology requires a probe containing an LED light source and a photodetector that are attached to the skin. The light source illuminates the tissue, and the photodetector senses the small variations in light intensity associated with changes in perfusion in the investigated volume. PPG can provide a large amount of real-time information about the cardiovascular system [14], such as blood pressure, heart rate, and oxygen saturation [15,16]. It is one of the most significant techniques in patient monitoring and pulse oximetry, even becoming mandatory according to the international standard for monitoring during anesthesia [17]. However, there are several limitations associated with conventional PPG, including lack of proper measurements on damaged skin, such as burned skin or wounds, ulcer or traumas, with consequent poor assessment of skin healing. Furthermore, since the contact PPG sensor should stay in contact with the skin, measurements become impractical on areas where free movements are required. In addition to that, force caused by contact of the PPG device may also lead to deformation of the microcirculatory vessel walls in the investigated region, thereby disrupting the microcirculation and causing discomfort to the patient. Furthermore, as a spot measurement, only pulsation monitoring in a localized small region is possible. All these issues can be solved by implementing this technique on a camera (imaging PPG) for remote detection of the PPG pulsation wave.

Imaging PPG (iPPG) is a novel technique that offers the same capabilities as contact PPG [18]. Recent studies showed the great potential of iPPG in perfusion assessment in various clinical applications [19,20,21]. Unlike contact PPG that requires skin contact with an optical sensor—thereby necessitating a pinpoint area of evaluation [9,14,15,16,17]—iPPG utilizes an off-the-shelf high-end camera and a light source. The dynamic changes in blood volume at the surface of the skin are remotely detected, allowing extraction of blood pulsation signals [22]. In this way, imaging PPG overcomes the issues of contact PPG mentioned above. Furthermore, a larger area of the skin can be imaged at once, allowing for spatial evaluation of the microcirculatory perfusion, defined as blood volume changes over time and quantified as the pulsatility of the PPG signal. Despite all its advantages, it is generally known that remote sensing approaches are more vulnerable to motion artifacts than the corresponding sensors in ordinary contact PPG devices. Additionally, iPPG can be affected by ambient light conditions, whereas contact PPG devices are relatively shielded from the environment. Furthermore, light source-detector separation decreases light penetration depth and quantitative measurements can be less accurate with iPPG. Various algorithms have been proposed for contactless iPPG measurements, of which most of these aim to substitute contact PPG, measuring vital sign parameters [23,24,25]. Extensive studies have been carried out for extracting and enhancing the heart rate from videos of the face or hands [26,27,28], and even a method for blood pressure measurements has been developed for iPPG systems [25]. Instead of simply acquiring spot measurements, iPPG allows for the elaboration of large tissue areas, thus building a so-called perfusion map, which is a great advantage with respect to contact PPG [29]. As Lai et al. showed, iPPG perfusion maps are capable of detecting skin perfusion perturbations, such as skin irritation, temperature changes, and even flow blockage during pressure measurements [30], also showing the potential of this technology for peripheral arterial disease assessment [31].

Imaging PPG can be compared to another technique for monitoring skin perfusion, the Laser Speckle Contrast Analysis (LASCA) [32,33]. While imaging PPG exploits the color changes of the pixels for extracting the perfusion, LASCA exploits the random interference effect that gives a grainy appearance to objects illuminated by a diverged laser light. In the case of light scattered from a large number of individual moving scatterers, such as particles in a fluid like blood, the speckle pattern fluctuates. The fluctuation correlates with the velocity distribution of the scatterers, and a map of the perfusion is produced. For LASCA, the targeted body part needs to remain still while recording, since the motion of the body affects the speckle pattern at the same way of the blood cells in the capillary bed, and the two motions cannot be separated. However, tissue stabilization is required just for a short amount of time, since a map of perfusion can be processed from a single speckle image acquired on the tissue. Moreover, several steps towards the use of this technology as quantitative perfusion method have been made recently by exploiting multi-exposure times, even though further exploration is still required [34].

With respect to LASCA, iPPG can be implemented by simply using a light source and a camera, but only a qualitative evaluation of the tissue perfusion is possible at the moment. Furthermore, iPPG requires a much higher processing time, since blood pulsatility is extracted from a video of at least 5 to 10 s. An advantage of iPPG over LASCA is that the blood pulsatility can be separated from the tissue motion by implementing motion stabilization algorithm, prior to iPPG maps extraction. Motion artifacts can be further suppressed by using PPG algorithms that combine video data captured at different wavelengths. An example of this is the Plane-Orthogonal-to-Skin (POS) algorithm that combines PPG signals extracted from the red, green and blue channels of an RGB camera [35]. However, in challenging situations, the motion requires further compensation, such as with tissue deformation.

Based on the great advantages that this technology has shown for remotely and non-invasively assessing skin-level perfusion, iPPG can potentially be translated to tissue perfusion assessment for detecting the perfusion of the microvasculature tissue bed at the organ surface in real-time. Introduction of iPPG in intestinal surgeries would allow for direct feedback to the surgeons of the perfusion status of the anastomosis, thereby enabling timely adjustments in the surgical plan when perfusion proves to be inadequate. Naturally, the translation of this technology from skin to organ perfusion brings new challenges to overcome. Motion and noise are factors that can mask or cover the iPPG signal, thereby compromising the typical PPG modulated wave, and therefore need to be reduced or completely removed. Areas of recording for iPPG on the skin are mainly the face, the palm of the hand and the plant of the foot. Therefore, the motion to be compensated for is mainly translational, rotational and scalar, and skin deformation is negligible due to the presence of connective tissue, muscles and bones underneath the skin. On the other hand, organs introduce the deformation as a further source of motion in which breathing and heart beating movements propagate towards the organs’ soft tissue, thereby increasing the level of motion. Furthermore, experiments on skin perfusion are carried out in experimental lab settings, with controlled conditions that reduce noise and variability, such as stable illumination, fixed distance to the target tissue, and preset camera settings. On the contrary, the operating theatres introduce an additional level of complexity. First, a certain distance from the sterile area of the patient needs to be maintained. Secondly, different arrangements of illumination and camera positions are necessary to address the different target areas inside the body, and thirdly, general unpredictable patient conditions need to be addressed. Therefore, new strategies need to be implemented to compensate for the motion and noise on the recorded tissue in the new experimental environment of the operating room.

In this study, we aim at evaluating a novel iPPG acquisition system with off-the shelf hardware for detecting the perfusion of the microvascular tissue bed at the organ surface of small intestines, thereby allowing for differentiating between perfused and non-perfused areas. Tissue perfusion levels are assessed at several surgical steps, on perfused and non-perfused intestine tissues, as well as after performing the anastomosis. The proposed PPG imaging setup enables the implementation of the POS algorithm for motion artifacts suppression, combining video data captured at different wavelengths. Besides PPG amplitude maps, additional parameters are extracted to provide a more detailed description of the perfusion status of the tissue. Strategies for limiting motion and noise during acquisitions were implemented and broadly discussed, providing a proof of concept for the use of iPPG for organ perfusion assessment during intestinal surgery.

## 2. Materials and Methods

### 2.1. Study Design

To explain the iPPG technique from data acquisition to data analysis and results, three datasets were used from small intestinal surgeries which display typical acquisitions and results. All patients underwent an open resection of a part of the small intestine in order to remove a tumor, after which the continuity of the intestines was restored with a side-to-side anastomosis. The study was approved by the Institutional Review Board (IRB) of the Antoni van Leeuwenhoek—The Netherlands Cancer Institute (AVL-NKI) hospital and registered under number IRBd19-155. Written informed consent for participation was signed by the patients included in the study. Data was acquired according the IRB guidelines of the AVL-NKI.

### 2.2. Data Acquisition

**Figure 1:**
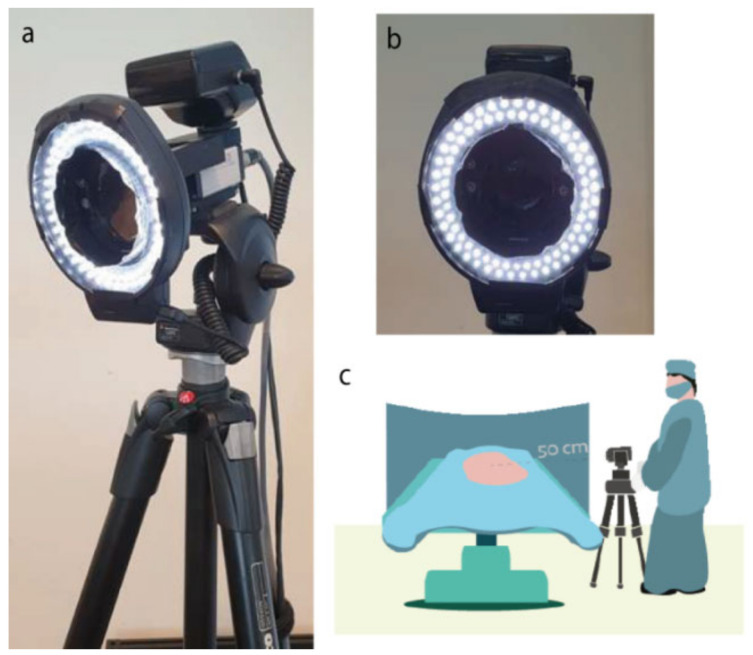
(**a**,**b**) The iPPG setup consisted of an RGB camera equipped with a Pentax-A 52 mm lens and a LED ring. The entire setup is placed on a tripod which allows the camera to be placed in close proximity to the patient during the surgical procedure. A cross-polarizing filter removes specular reflection and allows diffuse reflection to reach the lens. The camera was connected to a laptop via a Gigabit Ethernet outlet. (**c**) During acquisition, the camera was positioned approximately 50 cm from the target tissue. Camera settings were adjusted manually.

The iPPG setup was composed of a 2.8-Megapixels RGB camera (Manta G283B, Allied Vision Technologies GmbH, Stadtroda, Germany), which mounted a Pentax-A 52 mm lens (Max. aperture F1.4, Pentax, Tokio, Japan) and a LED ring (Falcon Eyes Macro ring-light MRC-80FV, Benèl BV, Hoogeveen, The Netherlands) equipped with a cross-polarized light filter (Edmund Optics, Visible linear polarizing laminated film, extinction ratio 44:1, wavelength range 450–675 nm). The entire setup of camera, lens and LED ring was mounted on a tripod (Manfrotto 055XDB + 128RC, Vitec Imaging Solutions Spa, Cassola, Italy) and is shown in Figure 1.

The spectral response of the camera covers the visible range, from 400 to 700 nm, with maximum quantum efficiency (QE) of the blue channel of 45% at 450 nm, QE of the green channel of 50% at 540 nm, and QE of the red channel of 50% at 600 nm. The spectral response of the camera and its specifications can be found in the datasheet. The LEDs in the ring have a color temperature of 5000 K, with their emission spectrum showing a peak in the blue range with a further hump in the green and red region. The spectral response of the camera matches the emission spectrum of the LED light. The LEDs are continuous-wave (CW) operated, with a battery pack that provides power to the LED ring. The LEDs are turned on during each acquisition, with its light intensity well uniform along that period of time. Furthermore, the objective of the camera is centered in the LED ring, allowing achieving a uniform tissue illumination, with light coming from all the directions and avoiding shadows on the tissue. The cross-polarized filter allows for removing the specular reflection, since only the diffuse reflection interacted with the tissue and is required to extract the PPG signal. Videos of the tissue were acquired at the frame rate of 20 fps, and 12-bit color depth. The 12-bit color depth was chosen to minimize the quantization noise. Videos were saved as image frames in a lossless file format, specifically in the uncompressed TIFF format.

The iPPG camera was positioned at a distance of approximately 50 cm from the target tissue. Since the setup was placed in the sterile field, a sterile drape was used to cover the tripod. During acquisition, the LED ring was turned on, while all other lamps in the operating room were switched off to avoid any light interferences. The camera was connected to a laptop via a Gigabit Ethernet outlet, and the camera settings and video acquisition were controlled via a proprietary user interface written in LabVIEW (National Instruments, Austin, TX, USA). The user interface allows for recording at the maximum resolution of 968 × 728 pixels and for the control of exposure time and color gain, which are essentials for achieving good acquisitions for processing PPG images. The gamma of the image was left at its default value (gamma = 1.0). The user interface does not have preset white balance values (e.g., incandescent, daylight), and the camera attributes were left at their default (RatioSelectorRed = 1.5, RatioSelectorBlue = 1.5). With the lens set at its maximum aperture, optimal focus on the target tissue was obtained by manually adjusting the focus of the camera. With the current camera configuration, the field of view at 50 cm of distance from the target is approximately 9 × 7 cm, which translates in a spatial resolution of 0.094 mm/pixel. The exposure time and color gain were set to achieve an average pixel intensity of the target area of 3200/4095 and avoid saturation.

Exposure time was the first adjusted setting, thus to maximize the light that hits the sensor that brings the actual PPG signal. Only afterwards the color gain was adjusted. This is because the color gain controls the amplification of the color signal and therefore also the amplification of the quantization noise, which in turn reduces the shades of light in the image. The exposure time was increased until the pixel intensity reaches the level mentioned above up to 45 ms, which was just below the maximum achievable level of 50 ms at 20 fps. This was done while keeping the color gain still at 0. However, if the desired intensity was not reached the color gain could be increased to achieve optimal intensity.

Three videos of 30 s of the intestine were acquired at different stages during the surgical procedure. At the start, a video of a fully perfused healthy part of the bowel was acquired, which is used as a reference (*baseline*). Later in the procedure, a video was acquired when the vasculature for that part of the intestine was dissected (*no perfusion*). At the end of the procedure, a video of the anastomosis was acquired (*anastomosis*). The intestine was stabilized during the acquisition to minimize motion artifacts caused by breathing, heartbeat, or intestinal peristalsis of the patient.

### 2.3. Data Processing

iPPG utilizes an off-the-shelf camera and a light source to remotely detect the dynamic changes in capillary blood volume within tissue surfaces. The pulsatile PPG information is encoded in the recorded uncompressed video over time. Acquired videos were processed using Matlab R2019b (The MathWorks Inc., Natick, MA, USA). Blood pulsatility information is encoded in the video as subtle changes in pixel brightness. It is essential that each pixel looks always at the same portion of tissue, so that only the pulsatility information related to that specific tissue area is contained in the pixel. To achieve this, motion stabilization is implemented on the acquired intestine videos. First, portion of the intestine visible in all frames of the video was manually selected as a region of interest (ROI). Then, video stabilization based on the Kanade-Lucas-Tomasi (KLT) algorithm was applied to the region of interest before the PPG signal could be extracted [36]. PPG signals and PPG maps were extracted from the entire length of the stabilized video, which was 30 s. In case motion occurred at the initial or final part of the video, that part was discarded and a shorter portion of the stabilized video was processed, which was always at least 20 s. To further increase the signal-to-noise ratio (SNR), a low-pass filter on each video frame was performed using a 10 × 10 pixel convolution kernel. The selected kernel size allowed for the local increase of the SNR of each pixel, without compromising the final spatial resolution of the iPPG maps, which stayed in the order of millimeters (with the current camera parameters).

**Figure 2:**
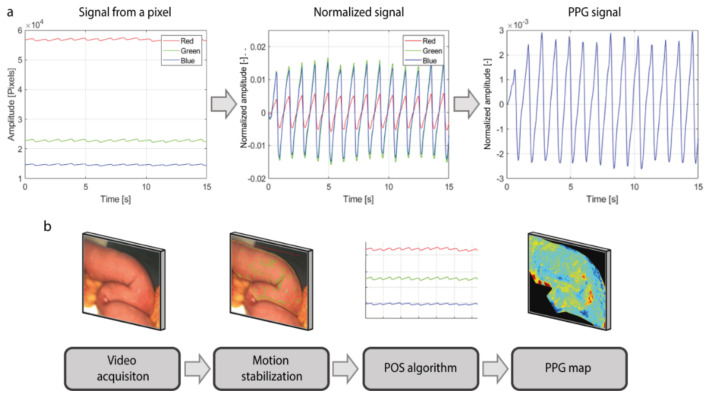
Workflow for data processing. (**a**) Plane-Orthogonal-to-Skin (POS) algorithm for PPG signal extraction. First, the PPG signals from the red, green, and blue channels of the video are extracted. Then, the pulsatile component AC of the signal of each pixel is normalized for its baseline color component DC. Afterwards, red, green, and blue signals from each pixel are combined. (**b**) Schematic overview of the workflow for building PPG maps of perfusion. After video acquisition, motion stabilization is applied to the region of interest. Afterwards, the POS algorithm is used to extract the PPG signal from each pixel and, eventually, the PPG maps are obtained using the Lock-in amplification algorithm.

The PPG signal was extracted from each pixel using the Plane-Orthogonal-to-Skin (POS) algorithm, which exploits the fact that green light is absorbed much more by the hemoglobin in the blood rather than red and blue light (Figure 2a) [35]. First, PPG signals from the red, green, and blue channels of the video were extracted. Then, the pulsatile component Alternating Coupling (AC) of the signal of each pixel was normalized for its baseline color component Direct Coupling (DC). This normalization expresses the amplitude of the PPG signal as a percentage and compensates for the parameters that affect both pulsation and baseline color level components, such as the intensity of the light or tissue color. The baseline DC component comprises frequencies up to 0.5 Hertz (Hz), whereas the pulsatile AC component comprises frequencies within the range of 0.5–10 Hz (30–600 bpm). Afterwards, the differences between the red, green and blue signals are computed and combined together, in order to enhance the PPG signal contained in the green channel, which is the channel that contains the stronger pulsatility component among the three. Furthermore, computing the differences between the signals allows for removing the noisy components, such as motion artifacts or light fluctuations, that affect the red, green, and blue signals in the same way. A detailed description of the POS algorithm can be found in the article of Wang et al. [35]. Subsequently, PPG maps containing the information about the amplitude of the signal and delay of the pulsatility were created following the Lock-In amplification method, as published previously [30,31]. The series of steps for building the iPPG maps of perfusion are shown in Figure 2b.

The computational time for PPG image processing depends on the video frame size, the length of the video, as well as to the used computer. With the current video size (968 × 728) and video length (30 s), PPG calculation takes an average of 3 min with our laptop (HP Zbook 15 G3, processor i7 8th Gen, RAM 32 Gb). However, the algorithm used for the computation is still under development and needs optimization. Motion stabilization and PPG signal and map processing, which are currently running on CPU, can be implemented on GPU to optimize and speed up the computation. Better performances will be achieved in the future, thus that PPG images will be processed in (near) real-time, while the patient is still in the surgery room. Based on our experience with the current algorithm and the advantages of using parallel computing on GPU to speed up the computational process, with an optimized algorithm, computation time is expected to be less than 30 s (with the same computer).

**Figure 3:**
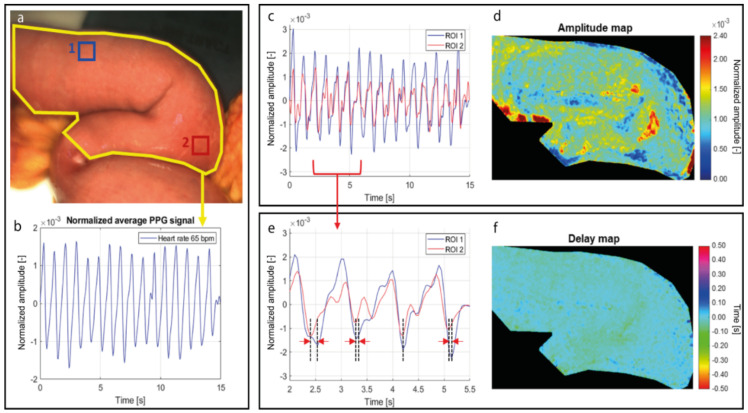
PPG signals and PPG maps extracted from videos. (**a**) RGB image of the recorded intestine area. The yellow contour represents the entire region of interest, the blue and red squares represent smaller, spatially different ROIs. (**b**) Global PPG signal extracted from the entire region of interest (yellow contour). (**c**) PPG signals extracted from ROI1 (blue) and ROI2 (red). (**d**) PPG amplitude map, which represents the amplitude of the PPG signal in each pixel. (**e**) Zoom-in of the PPG signals extracted from ROI1 and ROI2. The dotted lines indicate the variable time delay between each PPG wave arrival. (**f**) PPG delay map, which represents the average delay in the PPG wave arrival of each pixel, with respect to the global PPG signal.

By spatially averaging the pixel values in a predefined motion-stabilized ROI in the video (Figure 3a), the global PPG signal was extracted, which is a time-depend signal modulated at the heart rate (Figure 3b). The iPPG signal is contained in all the pixels of the video and can be extracted from any ROI selected (Figure 3c). These signals from spatially different regions are modulated at the same heart rate frequency although they have different amplitudes. By extracting the amplitude from the PPG signal of each pixel of the video and by color coding and assigning a red color to areas with higher perfusion and blue color to areas with lower perfusion, a PPG amplitude map was created, which represents the amplitude of the PPG signal per pixel (Figure 3d). 

Even though these signals were extracted simultaneously from the same tissue, the pulsation in each pixel can arrive at different moments. This is attributed to the blood pulsation arrival time, influenced by the microcirculatory bed resistance and elasticity of the vessels, as well as to different artery branches that supply the tissue, which is not always constant (zoom-in of PPG signals in Figure 3e). The delays of the PPG signals of each pixel, with respect to a reference signal, were extracted and used for building the delay map (Figure 3f). Since these delays are not constant due to small changes, as shown in Figure 3e, the delay map provides a measure of the average time delay between the PPG signal wave of each pixel and a reference PPG signal. With the current frame rate of 20 fps only delays multiple of 50 ms can be revealed, therefore only variations larger than 50 ms contribute to computing the average delay. Higher frame rates can be utilized for revealing even smaller variations of pulsatile changes.

In our study, the reference signal utilized for computing the delay map is the global PPG signal extracted from the entire region of interest. (Figure 3b). Since this reference signal was extracted from the entire ROI, a PPG signal from a given location of the image was likely to be synchronized with the reference. Similar to the map of the amplitude, also for the delay map each value is color-coded employing the hue saturation value (HSV) color system.

For each stage during surgery, the global PPG signal from the entire ROI, the frequency spectrum of the PPG signal, the amplitude map and delay map were extracted. Since the PPG signal is modulated at the heart rate frequency, the heart rate frequency corresponds to the highest peak of the spectrum. The remaining high peaks of the spectrum all correspond to the harmonics of the main peak. The SNR was extracted from the frequency spectrum and computed as the ratio between the area under the heart rate peak of the frequency spectrum and the area under the rest of the spectrum. For the SNR calculation, also the heart rate harmonics were classified as noise. The color scale of the PPG amplitude maps ranges from 0 and the maximum among the stages allowing for easy comparison of the perfusion levels. The color scale of the PPG delay maps ranges between ±0.5 s. Graphs were created and populated using single representative values for each PPG amplitude and delay map. For the PPG amplitude maps, the median value is used, whereas for the PPG delay maps the inter-quartile range (IQR) was used.

### 2.4. Data Analysis

The median of the amplitude map and IQR of the delay map of each acquisition were used to populate graphs displaying trends in intrapatient perfusion conditions. In addition, interpatient values were compared and assessed. The *baseline* conditions are deemed to be true-positive values of tissue perfusion, whereas *no perfusion* conditions are identified as true-negative values. Three patients are included in this feasibility study for the assessment of iPPG in a surgical setting. All iPPG videos for the individual patients acquired during the surgical procedure are included in this study. Due to this low number, a statistical analysis of the results is not included.

## 3. Results

**Figure 4:**
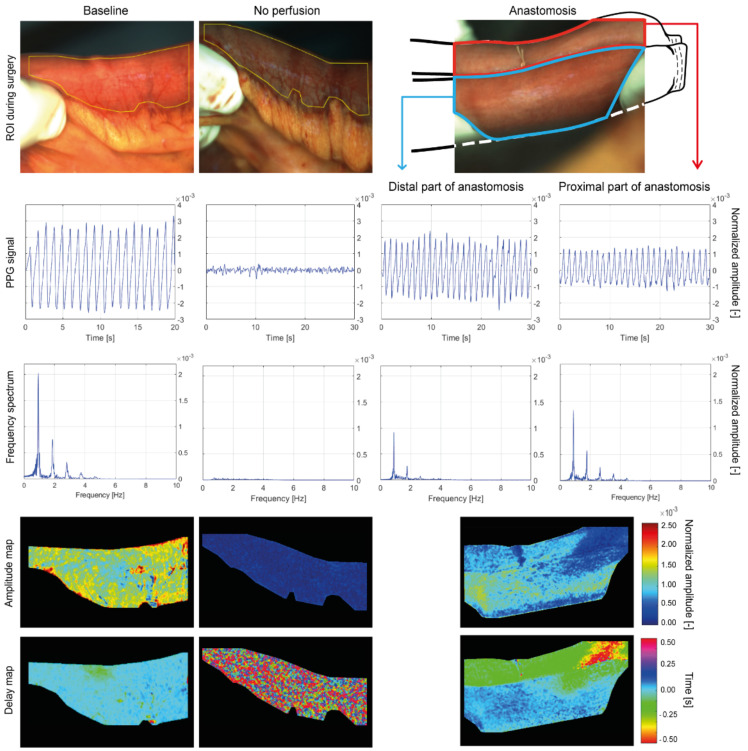
Analysis of the PPG signals and maps of the videos collected during intestinal surgery. The columns indicate the stages of surgery at which the videos were collected, namely *baseline*, *no perfusion*, and *anastomosis*. For the *baseline* stage, only 20 s of videos were recorded, instead of 30 s. The top row shows the RGB images of the intestine acquired during surgery, with the yellow contour indicating the ROI selected for processing. The ends of the intestines are stapled after removing the tumor and the continuity of the intestines was restored using a side-to-side anastomosis (schematic extension *baseline*, *anastomosis*). Distal and proximal ends of the anastomosis are processed separately. The second row shows the global PPG signal extracted from the yellow ROI. The third row shows the frequency spectrum of the PPG signal. The global PPG signal and the frequency spectrum of the distal and proximal ends of the anastomosis are processed separately. The fourth row displays the PPG amplitude maps, normalized with respect to the maximum among all the stages. The fifth row shows the PPG delay maps, normalized between −0.5 and 0.5 s.

Videos acquired from a patient that underwent intestinal surgery (patient 1) were analyzed and are presented here to provide a qualitative description of the processed data (Figure 4). This patient was chosen since it represents the typical dataset collected for this surgery. Three videos were acquired during an intestinal surgery (Figure 4, *ROI during surgery*). The global PPG signal from the *baseline* condition shows a clean pulsation level with little noise, whereas the signal of the *no perfusion* condition is flat and mainly consists of random noise. In the *anastomosis* stage, the proximal and distal parts of the intestine that were used to make the anastomosis are selected by the surgeon on the ROI image, and the global PPG amplitude was computed separately for each intestine end (Figure 4, *PPG signal*). The global PPG amplitude of the two intestine ends is different, with the distal part showing a higher amplitude, the proximal a slightly lower. However, both ends of the intestine show the high peak of the frequency spectrum at the same location, since both have been taken from the same video recording. In contrast, the *no perfusion* condition shows a spectrum with a frequency content several orders of magnitude lower than the other stages (Figure 4, *frequency spectrum*).

The PPG amplitude maps show good correlation to the perfusion state of the tissue; almost no PPG map amplitude during the *no perfusion* while higher PPG map amplitude in the *baseline* and *anastomosis* (Figure 4, *Amplitude map*). The delay map is more uniform in the *baseline* and the signal content in each pixel is coherent with the others, indicating that the pulsation arrives almost simultaneously in all the pixels, with variations due to resistance and elasticity of the vessels, motion artifacts, and uncertainty due to the chosen fame rate. For the *anastomosis,* the proximal and the distal ends show a different PPG time arrival, while still having a uniform delay within each of the two ends. Unlike the perfused conditions, the *no perfusion* map shows a random pattern of signal noise. Since the blood vessels of the intestine are ligated, the perfusion is completely absent, and the signal consists predominantly of incoherent signal noise. Therefore, the Lock-in amplification accounts for the random pattern of the delay map (Figure 4, *Delay map*).

**Figure 5:**
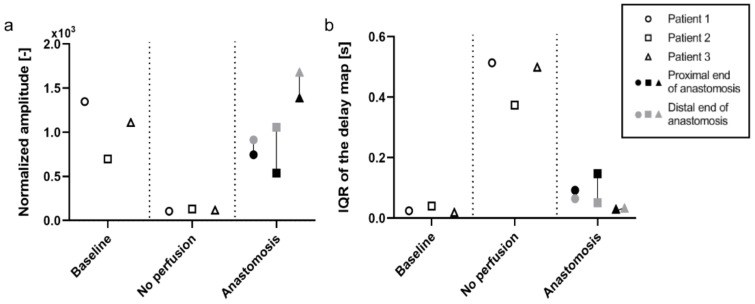
Graphs displaying iPPG trends of the amplitude and delay maps in different states of perfusion during surgery (*n* = 3). For the PPG amplitude maps the median value is used, whereas for the PPG delay maps the inter-quartile range (IQR) is used. (**a**) The median amplitude of the iPPG signal seems to be closely related to the perfusion state of the tissue, (**b**) whereas the IQR of the delay map seems to be inversely related to the perfusion of tissue. In the *anastomosis* condition, the reconnected bowel ends which form the anastomosis, proximal (black) and distal (grey), seem to be perfused differently.

Patient 2 and 3 were processed similarly to patient 1, and the median values of the amplitude maps and IQR of the delay maps were used to populate the graphs (Figure 5). The median of the amplitude map displays a similar trend between the three patients included. The PPG amplitude is high in *baseline* before dropping to almost zero when the vasculature is cut off. After the anastomosis is made, an increase in the signal is observed, comparable to the level at *baseline*. The distal end of the anastomosis shows a higher level of perfusion than the proximal end for all three patients (Figure 5a). The IQR of the PPG delay map gives an indication of the distribution of the PPG delay in the ROI. As said before, the delay map is very uniform when the pulsation arrives almost simultaneously in all the pixel areas, as is shown in *baseline*. When pulsation (PPG signal) is absent, as seen in the *no perfusion* condition, a random pattern appears, and the variability of the delay map increases. Consequently, there is a very low distribution for *baseline* and *anastomosis* (the delay is uniform in these conditions), while *no perfusion* shows a much larger and more variable IQR (Figure 5b).

**Figure 6:**
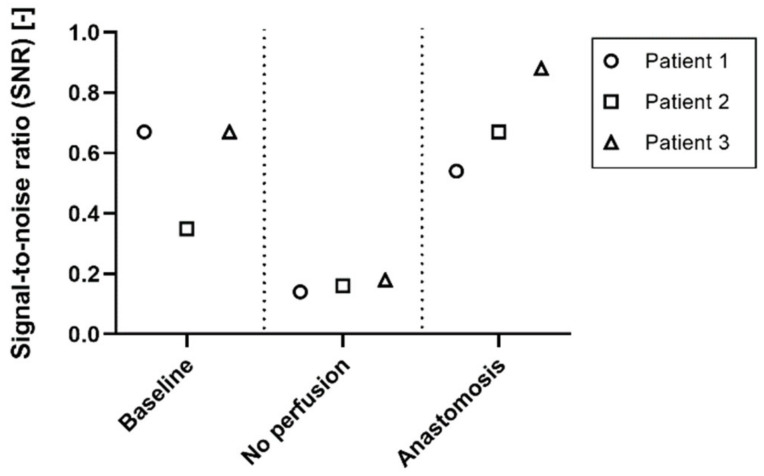
Signal-to-noise ratio (SNR). The SNR is low (<0.2) in the *no perfusion* conditions of all three patients, whereas it is much higher in the perfused conditions (>0.3; *baseline* and *anastomosis*).

The signal-to-noise ratio for the *baseline*, *no perfused* and *anastomosis* conditions of patient 1, 2 and 3 was used to identify the quality of the signal in the different stages of perfusion (Figure 6). The SNR changes between the perfused (*baseline* and *anastomosis*) and non-perfused (*no perfusion*) conditions. The SNR ranges between 0.35 and 0.67 in the *baseline* condition, and between 0.54 and 0.88 in the anastomosis. In the *no perfusion* condition, the SNR ranges between 0.14 and 0.18.

## 4. Discussion

Adequate tissue perfusion is of paramount importance to prevent postoperative complications [1]. Clinical examination and surgeons’ experience is the current golden standard to assess tissue perfusion intraoperatively [5]. Due to the lack of objective techniques to assess tissue perfusion in real-time during surgery, the need for such a technique is high. Imaging photoplethysmography (iPPG) is increasingly being researched for various clinical applications. For example, recently Kamshilin et al. showed the potential of PPG imaging for assessing cortical blood flow in brain surgery and anastomotic tissue perfusion in abdominal surgery [20,21]. In their study, PPG signals were acquired using a monochromatic camera setup and green light emitting LEDs. In this feasibility study, we utilized iPPG in an RGB camera with a spectral response of 400 to 700 nm for assessing tissue perfusion in a surgical setting. Three patients undergoing surgical resection of portion of the small intestine were included. This non-invasive approach could make real-time assessment of tissue perfusion in the future possible, thereby aiding in the prevention of postoperative complications such as anastomotic leakage.

The translation of the imaging PPG technology from skin to organ perfusion brings new challenges to overcome. New strategies need to be implemented to compensate for the motion and noise on the recorded organs in the new experimental environment of the surgery room. Besides the expected absence of signal in the not perfused tissue, several physiological factors and external sources of noise during acquisitions affected the quality of the PPG signal. Physiological factors could include temperature changes, which may lead to vasoconstriction, small regions of ischemic tissue or movement due to breathing, peristalsis, and even heartbeat-induced motion. 

It is of utmost importance to minimize, or at least mitigate, all the aforementioned sources of noise during video acquisition to increase the SNR and produce more reliable PPG maps. Since the noise can mask and cover the iPPG signal, compromising the typical PPG modulated wave, numerous measures are taken to reduce tissue motion. Peristaltic motion is relatively easy to be compensated. Since PPG signal and peristaltic motion do not stay in the same frequency range, peristalsis can be easily filtered out via signal processing. Furthermore, peristalsis is reduced due to administration of noradrenalin during surgery. Noradrenalin has an effect on the sympathetic nervous system, reducing digestive activity, and thereby reducing gastrointestinal motility. The most critical noise that needs to be minimized is the heart-induced motion. When the heart beats, the blood is pumped in the arteries and the motion wave due to the contraction propagates in the soft tissue. The heart-induced motion and the blood flow pulsation are at the same frequency (the heart rate) and because of this, the classic signal filtering techniques cannot be applied in this scenario. The most effective measure for this type of motion is achieved by lifting the targeted tissue to limit the propagation of the motion in the target area. This approach works well for limiting heart motion and breathing. This will be an additional advantage in surgeries where the tissues are fixated anatomically, as is the case in rectal surgery. Due to the retroperitoneal position of the rectum in the abdomen, the disturbances caused by external influences are minimized. As a result, clearer iPPG signals can be extracted. On the other hand, in case the noise induced by the heart motion is not properly compensated, iPPG signals might be extracted even from not perfused areas, resulting in an incorrect interpretation of the perfusion status of the tissue.

Factors that could reduce PPG signal quality are the difficulties in recording areas deep inside the body and the consequent low light illumination of those areas, sudden flashes of external surgical lights that may illuminate the tissue by mistakes, tremors, or malpositioning of the camera. The acquisition time for this study was set to 30 s of videos. This was a safe amount of time that enables the selection of a flexible window of at least 5 to 10 s continuously with neither illumination changes, movements of the surgeon, nor spontaneous tremors. From previous experiments on the skin, we learned that 5 s of acquisition was already sufficient for processing a PPG map, and in our publication on perfusion perturbations [30] we opted for acquiring videos of 10 s to be safe. As an example, even though videos of 20 s were recorded for the *baseline* stage (Figure 4), the PPG amplitude and delay maps processed were good.

Correct positioning of the camera and adjustment of the parameters, such as focus, exposure time, and color gain, is of high importance. Thus far, camera positioning and all camera parameters have been manually set by a skilled operator. A deep understanding of the iPPG technology is necessary to properly acquire videos that contain pulsatility information, and the process requires a learning curve. An automatic or semi-automatic method for setting the camera parameters would be required to facilitate the video acquisition for less skilled operators. While working in the sterile field in the operating room, tuning the necessary parameters, compensating for light illumination and different angles of acquisition, we always stayed at a safe distance from the patient, and disturbance of the surgical procedure was kept to a minimum. Lastly, the combination of the acquisition distance of about 50 cm, the 52 mm focal length of the camera objective, and the camera resolution of 968 × 728 pixels, allows for achieving processed PPG maps with optimal millimetric resolution. This enables the visualization of the detailed distribution of the perfusion status and allows for localization and identification of specific areas with compromised low perfusion.

An off-the-shelf camera and a light source were used to remotely detect the dynamic changes in blood volume in organ tissues during open surgery. We showed a striking difference in the amplitude of the PPG signal and PPG maps when comparing conditions in which blood flow is physiological and when blood flow is absent, which in turn translates to a difference in the level of perfusion. When the anastomosis is made, the blood flow in this newly made connection is comparable with the blood flow measured at the start of the surgery. Interestingly, we observed a difference in PPG amplitude within the proximal and distal parts of the intestine that were used to make the anastomosis. Furthermore, in the PPG delay map of *anastomosis* (Figure 4, *Delay map*), the proximal and distal parts of the intestine show a different PPG time arrival, while still having a uniform delay within each of the two ends. In the upper right of the *anastomosis* amplitude map, a portion of the intestine show a low PPG map amplitude that extends on both proximal and distal ends. The same portion of the intestine presents a PPG delay arrival of two different colors, green in the distal end and red in the proximal end (Figure 4). These phenomena can be caused by multiple factors. One factor can be attributed to the reperfusion after restoring the blood flow. Surgeons mobilize a portion of the intestine to excise the tumor. This mobilization of the mesentery causes vasoconstriction of the vessels due to mechanical manipulation. The perfusion rebounds after creating the anastomosis and releasing tension on the intestines, thereby causing a difference in perfusion between the proximal and distal ends of the anastomosis. Secondly, when an anastomosis is formed, two pieces of the intestine are placed in close proximity to each other whereas they are not under normal conditions. Therefore, they can be both supplied by blood with different arteries with different properties, which are physiological for that part of the intestine, but differ between different parts of the intestines. Another factor could be the anatomy of the superior mesenteric artery supplying the small intestine. This artery has multiple small branches supplying the different parts of the small intestine, and when a part is removed with the accompanying branches, the blood has to be divided over the remaining branches [37]. This may result in an increase in blood flowing through these vessels and can result in the perfusion differences that we observe in the anastomosis.

For all three patients included in this study, a high median value of the PPG amplitude maps was found for the *baseline* and the *anastomosis* conditions, whereas this is much lower for the *no perfused* conditions (Figure 5a). Despite lacking a statistical analysis, these results suggest that the amplitude of the PPG signal is associated with the perfusion status, and thus the flow of blood in tissues. A low IQR of the PPG delay map was found for the *baseline* and the *anastomosis* conditions since the pulsation arrives almost simultaneously in all the pixels in the ROI (high coherence). A high IQR is found in the *no perfused* conditions since the perfusion is completely absent and the signal consists predominantly of incoherent signal noise (Figure 5b). A uniform PPG delay map is correlated to a uniformed perfused tissue. Instead, randomly distributed delay maps are more correlated to no perfused tissue and can be used to further validate whether the target area is perfused or not. Secondly, areas with different PPG pulse arrival times on the same recorded tissue can be associated with different artery branches that supply the tissues. Therefore, the combination of PPG amplitude and delay maps give a better understanding of the distribution of flow in the tissue and thus perfusion status.

It is essential to assess the reliability of iPPG signals and distinguish recordings that show good signal quality from recordings that show poor quality, in order to better differentiate between no perfused and perfused tissues. The SNR of the *no perfused* conditions was always lower than all the perfused conditions, therefore the SNR could be used to define a threshold that separates perfused and not perfused tissue, giving an estimation of the reliability of the iPPG signals and iPPG maps (Figure 6). By identifying a threshold for the SNR, a quick separation can be made between reliable and unreliable iPPG signals. The results suggest that a SNR threshold between 0.20–0.30 will be sufficient to filter unreliable iPPG signals. However, the datasets need to be increased in order to estimate and define a proper threshold. Furthermore, the SNR was computed as the ratio between the area under the heart rate peak of the frequency spectrum and the area under the rest of the spectrum, where the heart rate harmonics are classified as noise too. A more advanced way of computing the SNR can be implemented in the future, that could also include harmonics in the iPPG signal. For example, instead of using the ratio, the SNR could be computed as the correlation between the acquired signals and a standard PPG signal used as template.

In this feasibility study, we described a method for detecting the perfusion status of intestinal tissues with a non-invasive iPPG setup, by processing data collected from 3 patients. These experiments show the great potential of translating iPPG towards organ perfusion monitoring during surgery. This objective imaging technique can potentially provide surgeons valuable feedback about the perfusion status of various tissues during surgical procedures. For example, in the case of intestinal anastomosis, a poor perfused anastomosis can be revised in the same surgical procedure to minimize the chance of perfusion-related postoperative complications. Thus far, only a qualitative evaluation of the technique has been provided, but in order to better understand and quantify the performances of iPPG for intestinal surgery, statistical analysis on a larger dataset is required. In this way, the clinical relevance of the perfusion maps and the iPPG signal as an objective perfusion indicator could be established and the predictive value of this method could be assessed. Additionally, comparing iPPG with imaging techniques that assess different perfusion parameters, such as HSI that acquires oxygen maps from tissues [8], will provide more detailed information about the intraoperative performance.

## 5. Conclusions

In this feasibility study, we evaluated an iPPG acquisition system for tissue perfusion assessment during intestinal surgery. New strategies were successfully implemented to compensate for the motion and noise on the recorded organs, in the new experimental environment of the surgery room. The experiments demonstrate that iPPG can be successfully used for extracting PPG signals and subsequently perfusion maps from the tissue surface, even detecting perturbations and perfusion changes during several stages of the surgery. Even though further exploration and more quantitative analysis are required on a larger patient sample, this skin perfusion measurement system can be potentially translated to organ perfusion monitoring during surgery.

## Data Availability

Data underlying the results presented in this paper are not publicly available at this time, but may be obtained from the authors upon reasonable request.
